# Atypical haemolytic uremic syndrome with refractory multiorgan involvement and heterozygous CFHR1/CFHR3 gene deletion

**DOI:** 10.1186/s12882-023-03153-x

**Published:** 2023-05-05

**Authors:** Jason Diep, Daniela Potter, Jun Mai, Danny Hsu

**Affiliations:** 1 Department of Renal Medicine, Liverpool Hospital, Locked Bag 7103, Liverpool, BC NSW 1871 Australia; 2 School of Clinical Medicine, University of New South Wales, Sydney, NSW Australia; 3 Department of Haematology, Liverpool Hospital, Liverpool, NSW Australia

**Keywords:** Case report, Thrombotic microangiopathy, Haemolytic uremic syndrome, Acute kidney injury, End-stage kidney disease, Kidney transplant

## Abstract

**Background:**

We present this challenging case report of Atypical Haemolytic Uremic Syndrome (aHUS) presenting with multi-organ involvement in a patient and heterozygous CFHR1/CFHR3 gene variant, which was refractory to initial eculizumab therapy.

**Case presentation:**

A forty-three year old female presented with aHUS and had heterozygous disease-associated deletions in the complement genes *CFHR1/CFHR3*. She had progressive kidney failure and severe extra-renal manifestations including cardiomyopathy and haemorrhagic cystitis; as well as pulmonary, gastrointestinal and neurological involvement. The initial kidney biopsy revealed thrombotic microangiopathy (TMA) changes involving all glomeruli. Clinical improvement was initially seen during eculizumab initiation with suppressed CH50 level, but a new rhinovirus/enterovirus upper respiratory tract infection triggered further severe multi-organ disease activity. The extra-renal manifestations stabilised, then ultimately improved after a period of eculizumab dose intensification. However, the impact on dose intensification on this improvement is unclear. Despite the extra-renal clinical improvement, she ultimately progressed to end-stage kidney disease (ESKD), commencing peritoneal dialysis for three years before undergoing a successful uncomplicated cadaveric kidney transplant without prophylactic eculizumab. Two years after transplant, she has excellent transplant graft function without any further disease recurrence.

**Conclusions:**

This case highlights the concept of extra-renal manifestations in aHUS initially resistant to eculizumab, which potentially responded to dose intensification. Whilst organ injuries are potentially reversible with timely targeted treatment, it appears that the kidneys are most vulnerable to injury.

## Background

Complement-mediated Atypical Haemolytic-Uremic Syndrome (aHUS) is a rare, life-threatening disease on the spectrum of thrombotic microangiopathy (TMA) disorders [1]. The classic clinical triad of aHUS is Coombs-negative haemolytic anaemia with fragmented erythrocytes (schistocytes), non-immune thrombocytopenia, and kidney impairment, all in the presence of normal ADAMST-13 activity [1]. Whilst thrombocytopenia can be mild or even absent in 15–20% of cases [2], there is often profound kidney injury, requiring initiation of renal replacement therapy in 81% of adults and 59% of children [3]. A kidney biopsy reveals histological features of TMA injury with thickened arterioles and capillaries, endothelial swelling and detachment, and intraluminal thrombosis with obstruction of the vessel lumen [1].

A genetic mutation affecting complement regulation can be identified in approximately 50–60% of complement-mediated aHUS [3, 4]. These mutations mainly involve the alternative complement pathway, resulting in either a gain-of-function of effector genes, or loss-of-function of regulatory genes [5]. This ultimately causes a state of chronic and relapsing complement system over-activation. Often an initial trigger of complement dysregulation is seen with each episode of aHUS, and can be due to multiple causes including viral or diarrhoeal illness, pregnancy, drugs, and solid organ or haematological stem cell transplants [1].

Initial TMA management includes therapeutic plasma exchange (TPE), until the differential diagnosis of thrombotic thrombocytopenic purpura (TTP) is excluded [2]. Eculizumab (Soliris, Alexion Pharmaceuticals), a humanised monoclonal antibody, inhibits complement dysregulation and is currently the treatment of choice for patients with aHUS [6]. It is not clear what the best treatment strategy is for patients whose disease initially does not respond to eculizumab.

## Case presentation

A forty-three year-old female presented with a one-day history of nausea, vomiting, and seven episodes of diarrhoea with lower abdominal pain. She also reported two-week history of fatigue, exertional dyspnoea, and a dry cough. She also noted paroxysmal nocturnal dyspnoea, bilateral lower limb oedema, and facial oedema. On retrospective collateral history, there was also subtle difficulties with concentration and memory.

Her significant medical history included relapsing remitting multiple sclerosis, with previous autologous stem cell transplant (fludarabine and cyclophosphamide conditioning) three months earlier. In the past, she had also had prophylactic bilateral mastectomy and oophorectomy for a known *BRCA* gene mutation with family history of breast cancer; two uncomplicated pregnancies; endometriosis; post-traumatic stress disorder; and generalised anxiety disorder.

Her medications on admission included aciclovir (CMV prophylaxis) and nebulised pentamidine (PJP prophylaxis) in the post-autologous stem cell transplantation period, gabapentin and diazepam for generalised anxiety disorder, and Progynova for hormone replacement therapy.

On examination, temperature was 36.7 °C, blood pressure 160/95 mmHg, pulse rate 90 beats per minute, respiratory rate 24 breaths per minute, and oxygen saturation 97% on ambient air. Examination was largely unremarkable other than mild non-pitting oedema in both ankles. Bedside urinalysis revealed mild blood and leukocytes.

Initial investigations revealed a newly raised creatinine of 142 μmol/L with an eGFR 39 mL/min/1.73m^2^ (baseline creatinine of 80 μmol/L and eGFR 79 mL/min/1.73m^2^, a year earlier). Her haemoglobin was 90 g/L (baseline 103 g/L, a month prior to presentation), platelet count 82 × 10^9^/L (baseline 164 × 10^9^/L), and white cell count 6.5 × 10^9^/L. Tests of liver function and coagulation profile were normal. Random urine protein-to-creatinine ratio was 206.9 mg/mmol, and there were no dysmorphic red blood cells, casts, or crystals.

On Day 3, High-Resolution Chest CT (HRCT) showed multifocal consolidation in the right lower lobe of lung. She was initially managed on empirical antibiotics with intravenous piperacillin/tazobactam and gentamicin, as well as escalating to intravenous acyclovir. She eventually escalated to intravenous voriconazole and meropenem, with only mild response to her symptoms. All investigations were unable to culture any micro-organism, including from sputum, stool, multiple blood cultures, CMV PCR, and bronchiolar lavage.

**Fig. 1:**
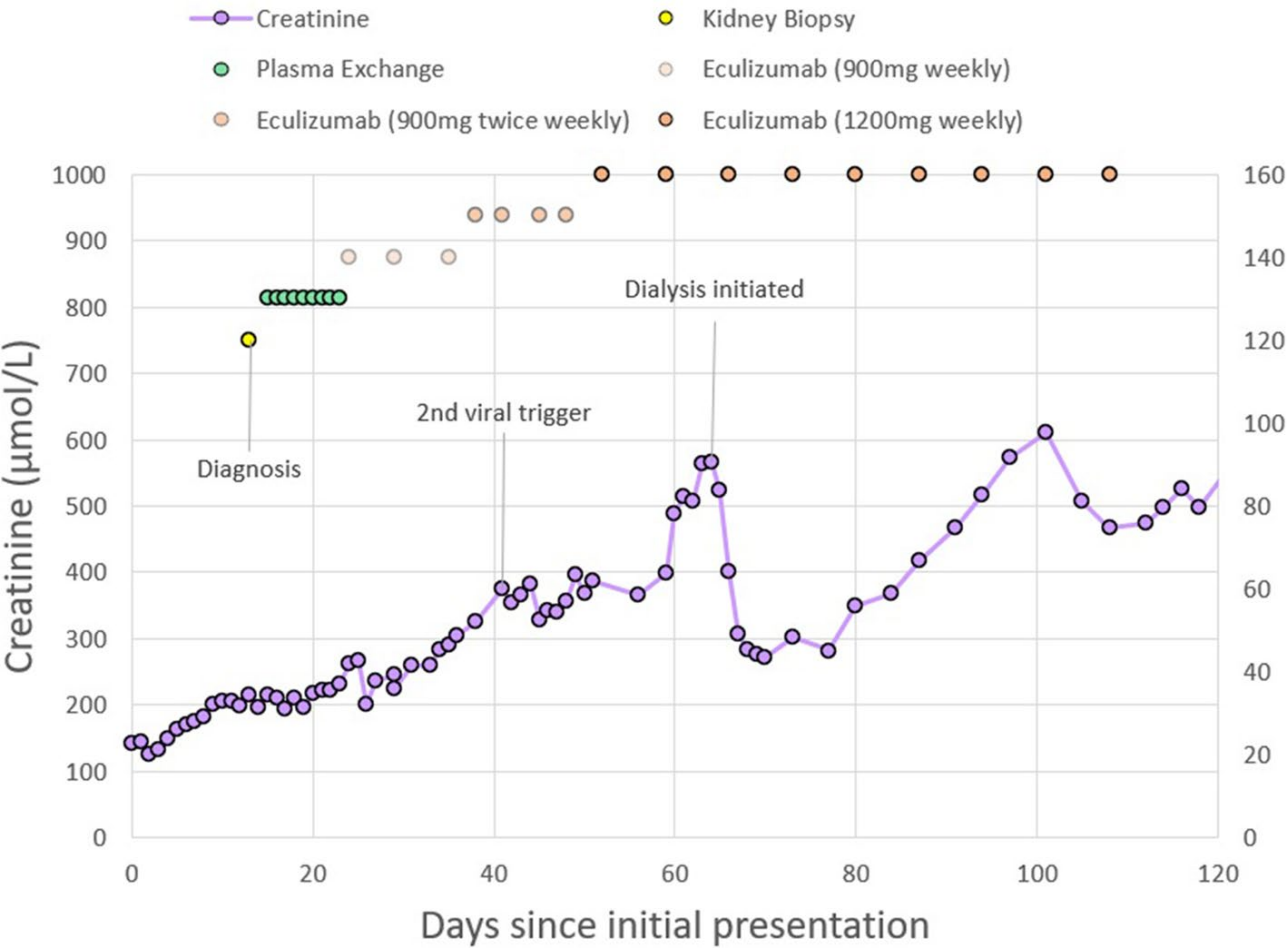
Progress of patient's kidney function from initial admission, management, and relapse

**Fig. 2:**
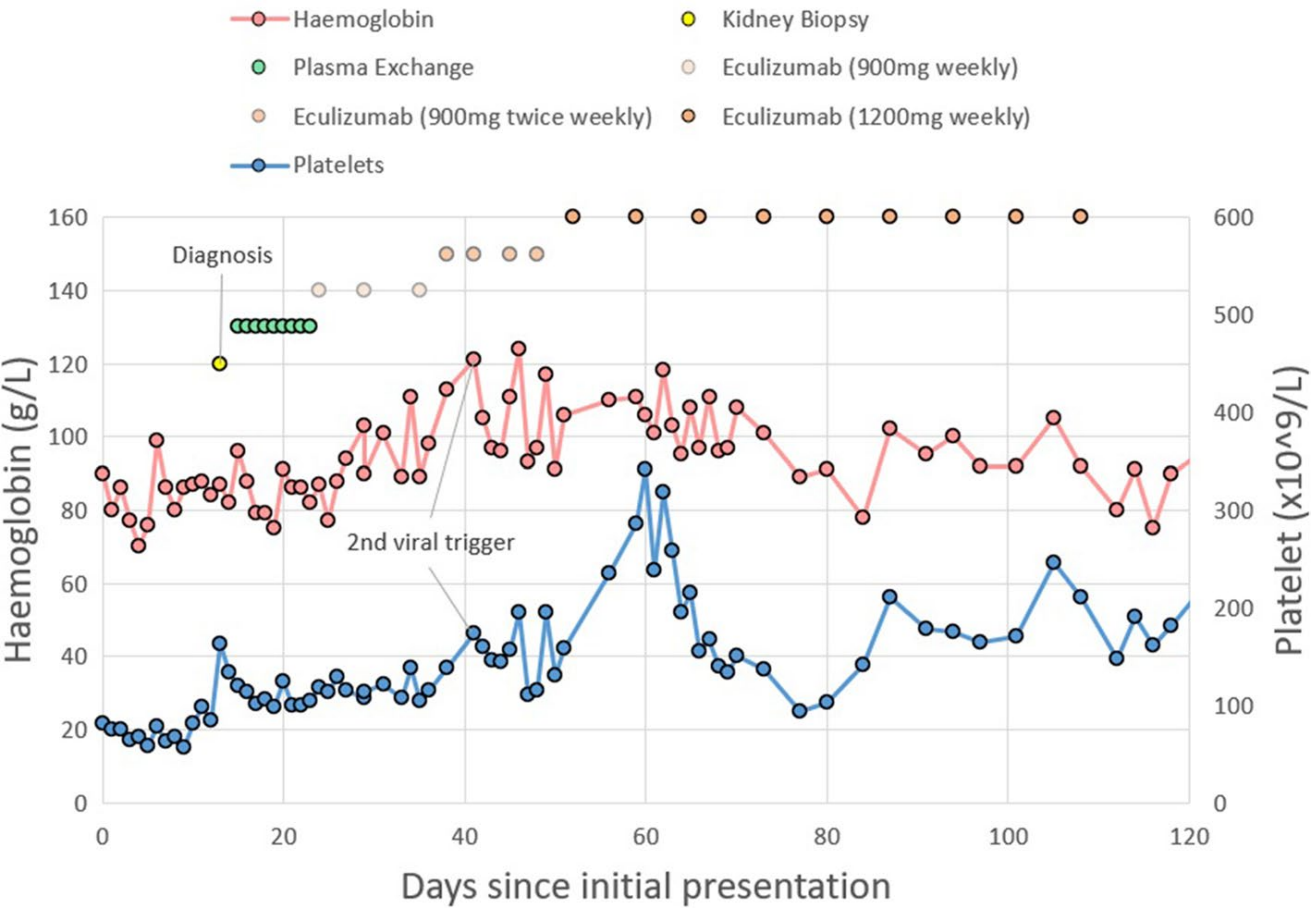
Progress of patient's haemolysis and thrombocytopenia from initial admission, management, and relapse

Her kidney function continued to decline to a creatinine 266 μmol/L and eGFR 18 mL/min/1.73m^2^, despite usual supportive measures (Fig. 1). A 24-h urinary excreted protein was 1.72 g/day, with urine volume 1500 mL. Autoimmune, vasculitis, and glomerulonephritis screen were all negative. From Day 4 of admission, there was a deterioration in her haemoglobin to 70 g/L, and platelet count to 67 × 10^9^/L, with red cell fragments detected on blood film (Fig. 2). Her haptoglobin was undetectable at < 0.08 g/L, with a raised reticulocytes to 288 × 10^9^/L, and raised LDH to 529units/L.

On Day 13, a kidney biopsy showed marked mesangial expansion with mesangiolysis of the glomeruli, with some showing foci of fibrinoid necrosis and capillary thrombi. The renal arterioles showed fibrinoid necrosis and subendothelial expansion. The diagnosis of thrombotic microangiopathy (TMA) was made.

Throughout this period, she developed persistent pulmonary oedema and bilateral pleural effusions. On Day 8, a transthoracic echocardiogram revealed moderate global systolic dysfunction (LVEF 40%) with mild-moderate mitral regurgitation. This would later deteriorate to moderate-severe segmental systolic left ventricular dysfunction (LVEF 35%) and severe systolic right ventricular dysfunction, along with severe pulmonary hypertension (pulmonary systolic pressure 53 mmHg).

She was commenced on TPE from Day 15–23 of admission, but despite initial rapid improvement in LDH, there was no improvement in her anaemia, thrombocytopenia, haptoglobin, and kidney function. Stool shiga toxin was negative. ADAMTS-13 activity was 58%. Complement studies showed C5 > 200 mg/mL, C6 100 mg/L, C7 110 mg/L, and C9 500 mg/L, C3 1.17 g/L, C4 0.25 g/L (Table 1). Complement Factor I (CFI) and Complement Factor H (CFH) were in the normal range. Genetic analysis was performed using Next Generation Sequencing (NGS) targeting entire coding regions of *ADAMTS13, C3, CD46, CFB, CFH, CFHR1, CFHR2, CFHR3, CFHR5, CFI, DGKE, MMACHC, PIGA*, and *THBD* genes; including ten base pairs of intronic flanking sequences. The NGS panel did not detect the presence of pathogenic variants. Multiplex ligation-dependent probe amplification (MLPA) was performed using SALSA® MLPA probe mix P236-A3 (provided by MRC-Holland), which detected heterozygous deletion encompassing the entire *CFHR1* and *CFHR3* genes. Soluble C5b9 and anti-CFH antibody testing was not yet available at the time of presentation in our institution.

**Table 1:** Complement study results

**Test**	**Value**	**Normal Range**
ADAMTS-13 activity	58%	40–130%
C3	1.17 g/L	0.79–1.52 g/L
C4	0.25 g/L	0.16–0.38 g/L
C5	> 200 mg/mL	100–169 mg/mL
C6	100 mg/L	45–96 mg/L
C7	110 mg/L	55–85 mg/L
C9	500 mg/L	125–265 mg/L

The patient was diagnosed with atypical haemolytic-uremic syndrome (aHUS). As well as haematological and kidney manifestations, it was suspected her disease demonstrated cardiac, respiratory, gastrointestinal, and possibly neurological involvement. We postulate that the initial infection triggered complement over-amplification in the presence of potentially contributory gene variants based on the combination HRCT findings, temporal distance from other background risk factors (cancer, surgery, stem cell transplant, chemotherapy medications, pregnancies), high C5 levels, and heterozygous CFHR1/CFHR3 gene deletions.

On Day 24, she commenced anti-complement therapy with Eculizumab 900 mg weekly infusion (along with Meningococcal B and ACWY vaccines, and amoxycillin 250 mg BD for two weeks), with some initial improvement to her anaemia, thrombocytopenia, and kidney function.

**Fig. 3:**
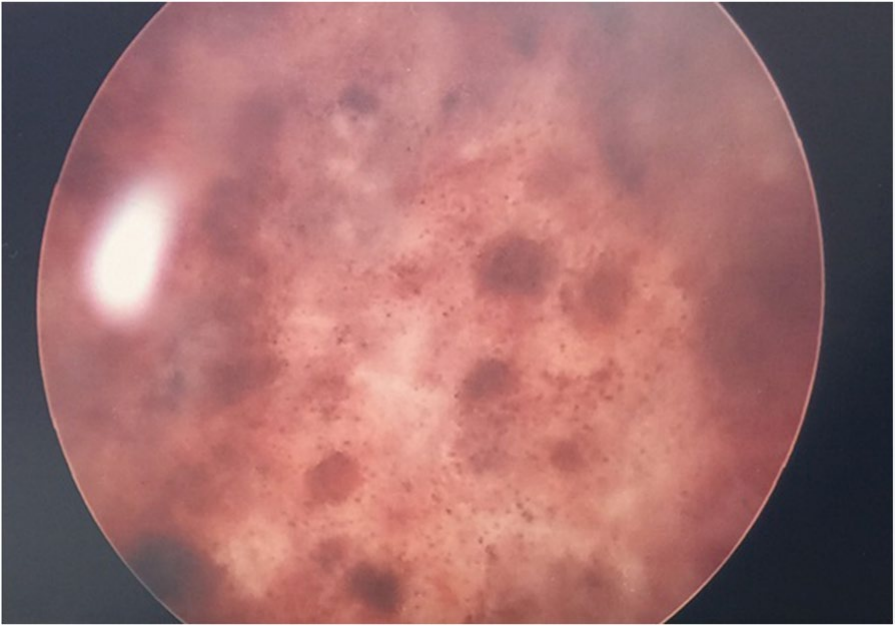
Cystoscopy of bladder reveals haemorrhagic cystitis and petechiae from microthrombi

Despite three weeks on eculizumab, she developed new clinical features of haemorrhagic cystitis in association with a new rhinovirus/enterovirus upper respiratory tract infection. Cystoscopy-guided bladder biopsy demonstrated active TMA (Fig. 3). After intensification of eculizumab therapy to 900 mg twice weekly, it was observed that her haematuria greatly improved. CH50 was < 10% (undetectable), suggesting adequate complement suppression with eculizumab. However, persisting thrombocytopenia and ongoing cardiac failure in the presence of worsening left ventricular ejection fraction suggested refractory disease, prompting further eculizumab dose intensification to 1200 mg IV weekly.

Although CH50 level was already suppressed, eculizumab dose intensification was trialled in an attempt to control refractory disease. During this treatment period, her platelet count normalised by the third week, and stabilised during the period of eculizumab dose intensification to 1200 mg IV weekly through weeks 5–9. The haptoglobin normalised by the sixth week. Eventually, her other extra-renal (gastrointestinal, respiratory, neurological) manifestations finally improved, and by sixth week her left ventricular function normalised.

Despite extra-renal improvement (and after seven weeks of eculizumab therapy), her kidney function continued to deteriorate to a serum creatinine 565 μmol/L with eGFR 7 mL/min/1.73m^2^, and she was commenced on haemodialysis. Subsequent prognostic kidney biopsies on commencement of (and ten months into) haemodialysis revealed lesions of TMA, but now with widespread tubular interstitial fibrosis and tubular atrophy. Eventually the patient had her eculizumab infusions weaned to 1200 mg fortnightly, and completed two years of therapy. No further recurrence or progression of extra-renal aHUS was detected, and she converted to long-term peritoneal dialysis.

After three years on peritoneal dialysis, she proceeded to undergo a cadaveric kidney transplant. She was deemed low risk for disease recurrence based on genetic findings and did not qualify for prophylactic eculizumab. Anti-CFH auto-antibodies were repeated pre- and immediately post-transplant, which remained negative. At two years post-transplant, her progress has been uncomplicated and without recurrence of aHUS or need for eculizumab therapy, with a recent serum creatinine 82 μmol/L with eGFR 74 mL/min/1.73m^2^.

## Discussion and conclusion

### Discussion

This was a challenging case of aHUS with suspected complement overactivation in which eculizumab dose intensification potentially led to improvements in her extra-renal manifestations, but unfortunately did not salvage her kidney function.

To our knowledge, this is the first reported case of biopsy-proven bladder involvement of aHUS, which eventually improved along with other multi-organ features. Secondly, this case raises a hypothesis that a new viral respiratory infection potentially caused additional complement amplification that overwhelmed the effect of standard-dose eculizumab. In aHUS, adequate eculizumab dose levels is suggested by CH50 activity < 10% or a eculizumab trough level > 100 mg/mL [2]. Similarly, in patients with paroxysmal nocturnal haemoglobinuria on eculizumab treatment, a CH50 < 10% is a surrogate marker of adequate plasma levels of the drug [7–9]. Interestingly, her CH50 was already found to be < 10% (undetectable) during the eculizumab 900 mg twice per week dosing period. We raise the potential hypothesis that her aHUS was complement-mediated, and that it required an intensified dosing regimen to achieve complete complement control.

The reason for the breakthrough aHUS activity despite adequate CH50 suppression is not clear. Cases of eculizumab “resistance” are extremely rare. In the paediatric post-bone marrow transplant setting, resistance may arise due to increased clearance of eculizumab, resulting in lower drug levels and incomplete suppression of CH50 [10]. However, this patient had successful suppression of CH50, and thus appropriate drug levels. The only other known mechanism of resistance is a rare C5 Arg885 variant, which can affect binding of eculizumab to C5 [11]. However, this variant is almost exclusively found in patients of Japanese and Hans Chinese ethnicity, which was not the case in this patient.

The question remains why her disease was resistant to eculizumab. A possible rationale for refractory disease despite suppressed CH50 has been described in paroxysmal nocturnal haemoglobinuria, another complement-mediated condition [12]. In the presence of intense complement activation, eculizumab (as an anti-C5 antibody) would be competing with de-novo C5 convertase (C3BbC3b) activity for free C5. Also, membrane-bound C5 convertases have higher affinity for C5 than fluid-phase C5 convertase [13]. During intense complement activation, higher concentrations of membrane-bound C3b increases its affinity to membrane-bound C5 convertase [14]. We postulate that in this patient, the second viral infection resulted in additional complement amplification with increased C3b deposition. This subsequently led to formation of high-affinity membrane-bound C5 convertase, which then displaces C5 from the eculizumab/C5 complex, allowing free C5 to trigger MAC formation [15]. In this way, we hypothesise the second viral trigger resulted in ‘breakthrough’ clinical TMA of the bladder. Ex-vivo measurements of CH50 levels remained suppressed throughout this period as expected, due to the presence of eculizumab. We propose that by increasing eculizumab concentration, it may have counteracted the membrane-bound C5 convertase’s displacement of eculizumab from free C5, achieving clinical response.

The patient presented in aHUS, just three months after an autologous stem cell transplant. Stem-cell transplant-associated TMA is a known complication, most often seen in allogenic transplants and results in poor outcomes [16]. It is most commonly reported in paediatric autologous stem cell transplants, likely due to higher exposure to chemotherapy and radiotherapy [17]. TMA is rarely reported in adult post-autologous stem cell transplants, and is more often associated with myeloablative conditioning regimens [18]. As her autologous stem cell transplant was three months prior to this presentation, it was felt that the initial complement-amplifying event triggering aHUS was the recent viral infection.

The patient has had several potential complement-amplifying events prior to this; such as two pregnancies, mastectomy and oophorectomy surgeries, and pre-conditioning chemotherapy; yet these did not result in any clinical manifestations of aHUS. On initial presentation, she had acquired hypogammaglobulinaemia with IgG of 4.4 g/L (6.39–15.6 g/L) reflecting delayed recovery of functional B-cells, and suggesting her immune system had not yet achieve full reconstitution from a functional perspective [19]. We postulate that in this post-autologous stem cell transplant immunocompromised state, the two infections likely triggered a complement storm which finally overcame her mildly deficient complement regulatory mechanisms. By the time she was about to receive a kidney transplant, her immune system had reconstituted, with an IgG 12.39 g/L (6.39–15.6 g/L). In the presence of heterozygous gene deletions, she had intact residual completement regulatory mechanisms, enough to counter the periods of complement amplification found in the kidney transplant surgery (and also the other surgeries and pregnancies).

This patient displayed the clinical phenotypes typical of a homozygous deletion of the *CFHR1* and *CFHR3* complement genes, which each have strong associations with anti-CFH auto-antibodies, cardiac manifestations, and overall worse prognosis [20]. However, her genetic testing revealed disease-associated heterozygous *CFHR1/CFHR3* deletions, which is less common but has been described in the literature [21]. In a cohort study of *CFHR1* and *CFHR3* deletions in aHUS patients, 35% demonstrated heterozygous deletions of *CFHR1* (versus 9% in control) [22]. Anti-CFH auto-antibodies have the strongest association with cardiac complications, being reported in 10% of cases, although mutations of *CFH*, *CFB*, and *C3* also are associated [20]. In one analysis of patients with anti-CFH auto-antibodies over three years, 63% reached ESKD or died, and only 12% had total remission of disease [23]. In the acute phase with anti-CFH auto-antibodies, 37% reached ESKD or died at one year [23]. Unfortunately, at the time of her initial presentation, anti-CFH antibody was not yet available in our laboratory. The precise overall underlying genetic contribution of heterozygous deletion to her clinical phenotype, and whether she had anti-CFH auto-antibodies at presentation, remains uncertain.

### Conclusion

aHUS has entered a new era as it becomes increasingly recognised and eculizumab becomes more accessible. However, there is still a high mortality rate, and patients that survive often remain dialysis-dependent. The challenge with aHUS is its heterogeneous constellation of overt organ involvement, where kidney dysfunction may not fully respond to therapy. The optimal timing, dose, frequency, and duration of eculizumab is difficult to determine in our patients given the significant limitations in laboratory monitoring of complement inhibitors. Each genetic mutation confers a different clinical phenotype and prognosis, clouding the clinician’s ability to forecast the outcome of kidney transplantation. Further research in this rare but debilitating condition could hopefully shed light on these issues.

## Data Availability

Not applicable.

## Abbreviations

aHUS: Atypical haemolytic uremic syndrome
CFB: Complement factor B
CFH: Complement factor H
CFHR: Complement factor H related protein
CFHI: Complement factor I
CMV: Cytomegalovirus
ESKD: End-stage kidney disease
eGFR: Estimated glomerular filtration rate
LVEF: Left Ventricular Ejection Fraction
MAC: Membrane attack complex
MCP: Membrane co-factor protein
PJP: Pneumocystis jirovii
TMA: Thrombotic microangiopathy
TPE: Therapeutic plasma exchange
TTP: Thrombotic thrombocytopenic purpura
